# Identification and quantification of immune infiltration landscape on therapy and prognosis in left- and right-sided colon cancer

**DOI:** 10.1007/s00262-021-03076-2

**Published:** 2021-10-16

**Authors:** Jun-Nan Guo, Du Chen, Shen-Hui Deng, Jia-Rong Huang, Jin-Xuan Song, Xiang-Yu Li, Bin-Bin Cui, Yan-Long Liu

**Affiliations:** 1 Department of Colorectal Surgery, Harbin Medical University Cancer Hospital, Harbin, 150086 People’s Republic of China; 2 The First Department of Oncological Surgery, The First People’s Hospital of Xiangtan City, Xiangtan, 411100 People’s Republic of China; 3 Department of Anesthesiology, The Fourth Affiliated Hospital of Harbin Medical University, Harbin, 150086 People’s Republic of China; 4 Department of Clinical Medicine, North Sichuan Medical College, Nanchong, 637000 People’s Republic of China

**Keywords:** Colon cancer, Left-sided, Right-sided, Immune cell infiltration (ICI), Prognosis, Therapeutic sensitivity

## Abstract

**Background:**

The left-sided and right-sided colon cancer (LCCs and RCCs, respectively) have unique molecular features and clinical heterogeneity. This study aimed to identify the characteristics of immune cell infiltration (ICI) subtypes for evaluating prognosis and therapeutic benefits.

**Methods:**

The independent gene datasets, corresponding somatic mutation and clinical information were collected from The Cancer Genome Atlas and Gene Expression Omnibus. The ICI contents were evaluated by “ESTIMATE” and “CIBERSORT.” We performed two computational algorithms to identify the ICI landscape related to prognosis and found the unique infiltration characteristics. Next, principal component analysis was conducted to construct ICI score based on three ICI patterns. We analyzed the correlation between ICI score and tumor mutation burden (TMB), and stratified patients into prognostic-related high- and low- ICI score groups (HSG and LSG, respectively). The role of ICI scores in the prediction of therapeutic benefits was investigated by "pRRophetic" and verified by Immunophenoscores (IPS) (TCIA database) and an independent immunotherapy cohort (IMvigor210). The key genes were preliminary screened by weighted gene co-expression network analysis based on ICI scores. And they were further identified at various levels, including single cell, protein and immunotherapy response. The predictive ability of ICI score for prognosis was also verified in IMvigor210 cohort.

**Results:**

The ICI features with a better prognosis were marked by high plasma cells, dendritic cells and mast cells, low memory CD4^+^ T cells, M0 macrophages, M1 macrophages, as well as M2 macrophages. A high ICI score was characterized by an increased TMB and genomic instability related signaling pathways. The prognosis, sensitivities of targeted inhibitors and immunotherapy, IPS and expression of immune checkpoints were significantly different in HSG and LSG. The genes identified by ICI scores and various levels included CA2 and TSPAN1.

**Conclusion:**

The identification of ICI subtypes and ICI scores will help gain insights into the heterogeneity in LCC and RCC, and identify patients probably benefiting from treatments. ICI scores and the key genes could serve as an effective biomarker to predict prognosis and the sensitivity of immunotherapy.

**Supplementary Information:**

The online version contains supplementary material available at 10.1007/s00262-021-03076-2.

## Introduction

The increasing morbidity and mortality of colon carcinoma (CC) have arisen as crucial public health issues [1]. The clinical treatment and prognostic evaluation of CC have always been a hotspot. In 1990, Bufill highlighted the obvious differences in epidemiology, cytogenetics and molecular characteristics between proximal and distal CC [2]. Given the physiology and anatomy of the colon, colon cancer can be divided into left-sided colon carcinomas (LCCs) and right-sided colon carcinomas (RCCs) [2–4]. In such categories, the clinical heterogeneity of LCCs and RCCs has also been studied more specifically (e.g., metastasis, recurrence, prognosis, and sensitivity of treatment) [5–7].

As mentioned in the literature review, LCC patients benefit more from chemotherapies and targeted therapies and have a better prognosis. RCC patients do not respond well to conventional chemotherapies, but demonstrate more promising results with immunotherapies [8]. Patients with RCC were found to have different molecular biological tumor patterns and a poorer prognosis than patients with LCC [9]. The crucial role of primary sites in treatment decision-making has been progressively clarified. Around 2015, the "dispute between LCC and RCC" become one of the hot topics in CC. Tumor primary sites are considered an independent prognostic factor for CC in stage III/IV. The prognosis of RCC is significantly worse than that of LCC, which is not related to relevant treatments. Additionally, RCC acts as a negative predictor of EGFR-targeted therapy [10].

It is noteworthy that LCC and RCC are inconsistent in numerous aspects (e.g., embryonic origin, anatomical blood supply and clinical manifestations). The critical culprit could cause the difference in treatment response, and prognosis is the molecular biological characteristics [11, 12]. However, stratification by tumor cells, molecular pathways, mutation status and tumor gene expression only exhibit moderate predictive accuracy and limited clinical utility [13]. The development of tumors has been significantly associated with the immune system. Immunotherapy progressively becomes the developing direction of tumor therapy, which exhibits unparalleled advantages and survival benefits for numerous cancers [14]. In 2017 NCCN guidelines, Anti-PD-1 monoclonal, an immune checkpoint inhibitor, was initially recommended for treatment of end-stage CC with dMMR/MSI-H phenotype [10]. However, the proportion of dMMR tumors only takes up about 5–8% [15], only a small part of colon cancer patients can benefit from immunotherapy [6]. For other patients, novel molecular subtypes should be identified to evaluate prognosis and assess treatment responses.

It has been extensively reported that TAIM is of critical significance to tumor development and immunotherapy responses [16]. The density of infiltrating T lymphocytes in colon cancer acts as a reliable estimate for the risk of recurrence and prognosis [17, 18]. However, tumor-associated immune microenvironment (TAIM) contains a wide range of cellular components, and the identification of T lymphocytes cannot effectively represent the complex tumor immune environment. As indicated from the research on various cancers, the inhibitory TAIM characterized by infiltration of a series of immune cells and stromal cells critically impacts tumor proliferation, metastasis, recurrence, and immunotherapy resistance [19]. Thus far, the characteristics of immune cell infiltration (ICI) in LCCs and RCCs remain unclear. More insights into TAIM of them should be urgently gained to identify the ICI subtypes that cause the difference of prognosis, as an attempt to lay a basis for improving immunotherapy.

This study aimed to develop a method to identify the ICI subtypes of LCC and RCC, and also to quantify the ICI landscape. As the sequencing technology is leaping forward, people have been enabled to accurately evaluate the immune infiltration of tumors by using algorithms. In this study, “CIBERSORT” and “ESTIATE” were used to analyze two gene expression datasets from different high-throughput platforms, and a series of comprehensive analyses were conducted on ICI in LCCs and RCCs. The samples were clustered based on the content of immune cells, and differentially expressed genes (DEGs) were screened out among different ICI clusters. With the mentioned DEGs, all samples were re-clustered and divided into three gene clusters. These three clusters exhibited unique characteristics of ICI. Subsequently, a clusters-based ICI score was set to distinguish the prognostic-related high- and low-ICI score groups (HSG and LSG, respectively), and significant differences were identified in the tumor mutation burden (TMB), prognosis and sensitivity of treatments between two groups. Lastly, “WGCNA” and prognostic analysis were conducted to screen out the gene modules with the highest correlation with ICI score and key genes. On that basis, we attempted to evaluate the prognosis and assess the treatment sensitivity more accurately.

## Materials and methods

The flowchart of the whole study was presented in Supplementary Fig. 1.

### CC datasets and samples

In the present study, 629 colon adenocarcinoma (COAD) samples originated from The Cancer Genome Atlas (TCGA) (Data Release 24.0, Release Date: 7 May 2020, https://tcga-data.nci.nih.gov/tcga/) and Gene Expression Omnibus (GEO) (http://www.ncbi.nlm.nih.gov/geo/) (GSE103479). Other relevant data included somatic mutation information and clinical information. The inclusion criteria were as follows: (1) The primary tumor sites of all patients were in the left or right colon. The LCCs included tumor primary sites in cecum, ascending colon, as well as hepatic flexure. The RCCs covered tumor primary sites in splenic flexure, descending colon, sigmoid colon, as well as rectosigmoid junction. The study excluded patients’ tumor sites in transverse colon and rectum. (2) All patients must have complete follow-up information and RNA-seq data. The gene ID of the respective dataset was converted to the corresponding gene symbol by complying with the gene annotation package. Moreover, the expression profiles were all transformed into TPM (Millions of Transcripts Per Kilobase) for the combined analysis. The “ComBat” algorithm was applied to reduce the likelihood of batch effects from non-biological technical biases between different datasets [20]. The analysis excluded RNA that was undetectable in over 10% of the samples.

### Estimating of immune cell infiltration (ICI) and sample clustering

The R package “ESTIMATE” [21] and “CIBERSORT” [22] were adopted to estimate the immune score, stromal score and 22 types of ICI. The correlation between the ICI components was analyzed. Subsequently, according to ICI pattern, hierarchical agglomerative cluster was performed by R package “ConsensusClusterPlus” [23]. "ConsensusClusterPlus" adopts an algorithm to determine cluster count and membership based on stability evidence in the unsupervised analysis. This algorithm was repeated 1000 times to ensure the stability of clustering.

### DEGs related to ICI subtypes

To identify the DEGs related to ICI, all samples were clustered into different ICI subtypes. The DEGs among ICI subtypes were analyzed using the R package “Limma” [24] (|log2foldchange|> 1.5, false discovery rate (FDR) < 0.05).

### Clustering with DEGs, dimension reduction and construction of ICI score

For sample clustering at the genetic level, unsupervised clustering was conducted to cluster samples according to DEGs. Here, DEGs positively and negatively correlated with gene clusters were defined as ICI gene signatures A and B, respectively. Gene Ontology (GO) and Kyoto Encyclopedia of Genes and Genomes (KEGG) functional annotations analysis was conducted by R package "clusterProfiler" [25]. To reduce the noise or redundant genes, the dimensionality reduction for ICI gene signatures A and B was performed by using Boruta algorithm [26], and the feature genes were identified. Subsequently, principal component analysis (PCA) was conducted to extract the main component 1 from feature genes as the signature score in the respective ICI gene signature. Lastly, a method similar to the gene expression grade index [27] was adopted to define the ICI score of each patient: ICI score = ∑PCA1A-∑PCA1B. To identify the HSG and LSG related to prognosis, the cut-off value was determined by using R package “maxstat” [28]. Then, after the combination with other clinical factors, we used univariate and multivariate Cox proportional hazard regression analysis (COX) to verify the independent predictive effect of ICI score groups. The gene set enrichment analysis (GSEA) was conducted by using the Bioconductor package “fgsea” [29] with 10,00 permutations between HSG and LSG.

To investigate therapeutic sensitivity, the concentration causing 50% reduction growth (IC50) of targeted inhibitors (TIs) was calculated by R package "pRRophetic" [30] (e.g., vascular endothelial growth factor receptor 2 (VEGFR2), Hedgehog (HH) and Wnt inhibitors). Wilcoxon rank-sum test was performed to compare the IC50 difference between HSG and LSG. Furthermore, the differences were analyzed in gene expression of the 6 immunosuppressive checkpoints in HSG and LSG.

In addition, immunogenicity is determined by a variety of immune-related genes, including genes related with effector cells, immunosuppressive cells, MHC molecules and immune regulatory factors. By using machine learning, immunogenicity can be evaluated and quantified without bias. We downloaded the Immunophenoscores (IPS) of colon cancer patients from the TCIA database (https://tcia.at/) [31, 32]. To predict sensitivity of immunotherapy, we compared the IPS between HSG and LSG in different immunotherapy decisions.

Meanwhile, we conducted a comprehensive search on the gene expression profile of public immunotherapy cohort and selected metastatic urothelial tumors cohort (IMvigor210: http://research-pub.gene.com/IMvigor210CoreBiologies/) [33]. We preprocess the data according to the method in the R package "IMvigor210CoreBiologies" [33] provided by the author. The RNA-seq data were filtered and normalized by R package "edgeR" [34] and then transformed by voom in R package "limma" [24]. We also compiled the clinical information and treatment outcomes. Based on the mentioned method above, we calculated the ICI score of each sample in the cohort.

### Analysis of somatic alternation data

To identify the TMB of HSG and LSG, the total number of non-synonymous mutations was counted in the TCGA sample. The difference in TMB was analyzed between HSG and LSG. Moreover, the correlation between TMB and ICI score was analyzed. Lastly, R package "maftool" [35] was adopted to analyze and demonstrate the gene mutation patterns and frequencies in different groups. Furthermore, the difference of TMB in HSG and LSG was analyzed by performing chi-square test.

### Identification and comparison of key genes based on ICI score

Weighted gene co-expression network analysis (WGCNA) was conducted on DEGs and ICI scores using the R package "WGCNA" [36]. First, the power function was used to build the adjacency matrix (AM) of DEGs, and an appropriate power index was selected. Subsequently, the AM was altered to a topological overlap matrix. Lastly, the gene consensus modules were obtained and correlated with the ICI scores. The mRNAs in the modules with the highest correlation with ICI scores were used to conduct the prognostic analysis. The prognostic model was built by conducting multivariate COX with the R package “glmnet” [37]. Afterward, ROC curve and AUC were evaluated with the R package “survivalROC” [38]. DISNOR (https://disnor.uniroma2.it/) [39], a disease network open resource, was conducted to analyze the upstream and downstream genes and the protein interaction of key genes. Furthermore, protein–protein interaction (PPI) analysis was conducted with STRING (https://www.string-db.org) [40]. The Single Cell Expression Atlas (SCEA) database (https://www.ebi.ac.uk/gxa/sc/experiments/E-MTAB-8410/) [41] was used to explore the key genes of colon cancer in single-cell level under the project accession E-MTAB-8410 (https://www.ebi.ac.uk/arrayexpress/experiments/E-MTAB-8410/). SCEA allows researchers to gain a quick insight into the expression pattern of their gene of interest at the level of individual cells across different species. By setting the appropriate t-distributed stochastic neighbor embedding (t-SNE) perplexity score and the number of clusters (*k* value), t-SNE and the marker gene heat map were plotted. T-SNE plots are a useful way of visualizing highly complex data in a 2D space. *K* value shows the output of the Scanpy clustering algorithm [42]. Scanpy clusters cells into subgroups using the Louvain algorithm. The cell types were inferred by sequencing analysis based on gene expression profile. The analysis methods applied to the raw data to obtain the clustering and gene expression results were shown in the Supplementary Table 1.

To further verify and screen key genes at the protein level, we downloaded the proteomic cohort of TCGA-COAD samples from The Clinical Proteomic Tumor Analysis Consortium (CPTAC) (https://proteomics.cancer.gov/programs/cptac) [43], including 29 normal samples and 64 tumor samples. The differential expression analysis of these genes identified by ICI score was performed. The immunohistochemical staining images were obtained from The Human Protein Atlas project (https://www.proteinatlas.org/] [44] to verify the actual expression. In addition, we also analyzed the expression differences of these genes in different treatment outcome groups in IMvigor210 cohort.

### Statistical analyses

R software (version 3.6.3) was employed for all statistical analyses. The Wilcoxon test was performed to draw the comparison between two groups, and Kruskal–Wallis test was performed for over two groups. The Kaplan–Meier plotter was adopted to plot the prognostic survival curve for the subgroups, and the log rank test was performed to evaluate the differences with statistical significance. The chi-square test was performed to analyze the correlation between ICI score groups and the TMB, and Spearman analysis was conducted to calculate the correlation coefficient. *P* < 0.05 showed statistical significance.

## Results

### The landscape of ICI in LCC and RCC

**Fig. 1:**
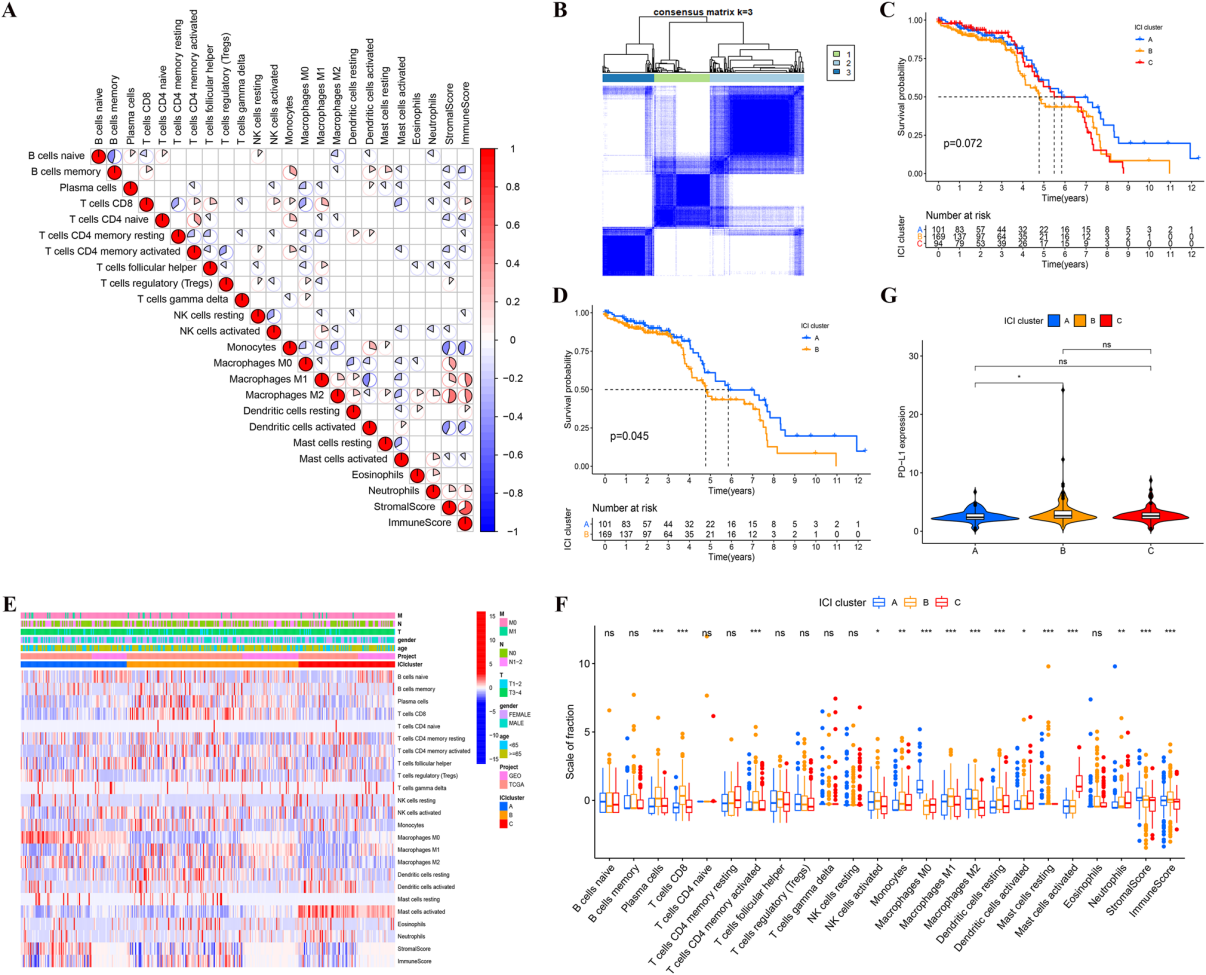
** A** The correlation heat map visualized the universal landscape of immune cell interaction in TME. The correlation coefficient decreased in size from red to blue. **B** Consensus matrixes of all CC samples for appropriate k value (*k* = 3), displaying the clustering stability using 1000 iterations of hierarchical clustering. All samples were clustered into 3 subtypes. **C** Kaplan–Meier curves of overall survival in different ICI clusters. Log rank test showed an overall *p* = 0.072. **D** Kaplan–Meier curves of overall survival between ICI cluster A and B. Log rank test showed an overall *p* = 0.045. **E** The heat map depicted unsupervised clustering of ICI in all CC samples. Rows represented tumor-infiltrating immune cells, and columns represented samples. **F** The fraction of tumor-infiltrating immune cells, immune score and stromal score in three ICI clusters. The statistical difference of three ICI clusters was compared by the Kruskal–Wallis test. **G** The difference of PD-L1 expression among distinct ICI clusters. (^*^p < 0.05, ^**^p < 0.01, ^***^p < 0.001, ^ns^*p* > 0.05)

By using the criteria, 411 LCCs and RCCs samples were included here (i.e., 322 samples from TCGA and 89 samples from GEO). The detailed information of 411 COAD patients was shown in Supplementary Table 2. We performed "ESTIMATE" and "CIBERSORT" algorithms to calculate the ICI content of tumor samples from TCGA and GEO databases (Supplementary Table 3 and 4). A heat map was drawn to visualize the interaction of immune cells (Fig. 1A), and the content of different types of macrophages was found to have highly positive correlation with immune score. Next, based on ICI content, the R package "ConsensusClusterPlus" was used to cluster all samples, and 3 subtypes were obtained (Fig. 1B) (Supplementary Table 5).

We compared the prognosis of these three ICI subtypes (Fig. 1C) and found that the prognosis of ICI cluster A was significantly better than that of ICI cluster B (*p* = 0.045) (Fig. 1D). To more specifically clarify the inherent differences of ICI that caused the prognostic difference, a differential analysis of immune cells was conducted in different ICI subtypes. The ICI cluster B related to worse prognosis was marked by high Plasma cells, CD8 + T cells, activated memory CD4 + T cells, M1 macrophages, M2 macrophages, resting dendritic cells (DCs), as well as low activated DCs and activated mast cells (MCs) (Fig. 1F). CD8 + T cells were found to be highly infiltrated in the poor prognosis subtype, so the expression differences of PD1 and PD-L1 were analyzed, which are important immune checkpoints of CD8 + T cells. As indicated from the results, the expression of PD-L1 in ICI cluster B was significantly higher than that in ICI cluster A (*p* < 0.05) (Fig. 1G).

### Clustering by DEGs from ICI subtypes

**Fig. 2:**
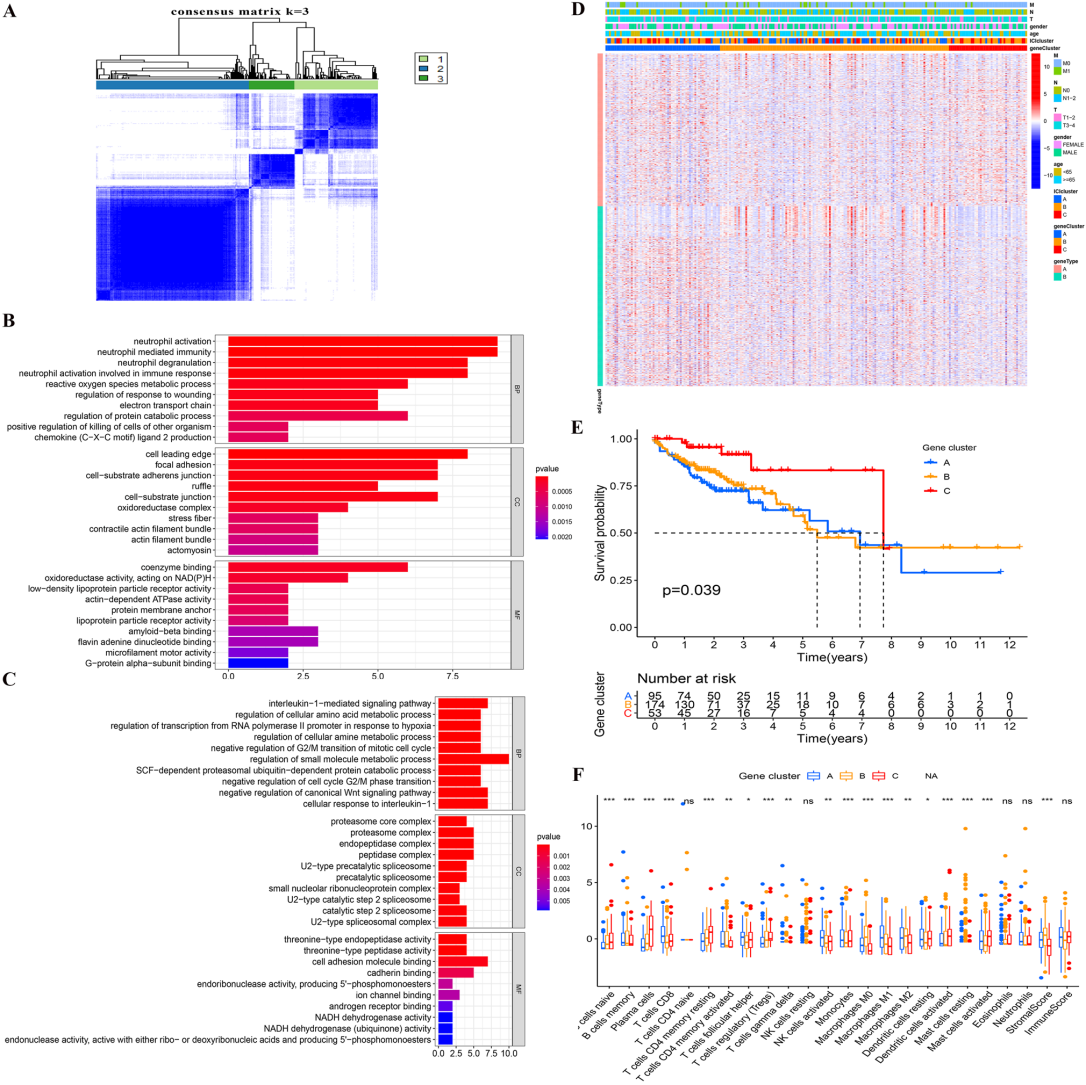
**| A** Consensus matrixes of TCGA-COAD cohorts for appropriate *k* value (*k* = 3), displaying the clustering stability using 1000 iterations of hierarchical clustering. TCGA samples were clustered into 3 subtypes based on the DEGs among three ICI clusters. **B** GO enrichment analysis of the ICI-relevant signature genes A. **C** GO enrichment analysis of the ICI-relevant signature genes B. The X axis indicated the number of genes within each GO term. **D** The heat map depicted the expression of DEGs in different ICI clusters and gene clusters. Heat map colors indicate relative DEGs expression levels. **E** Kaplan–Meier curves of overall survival in different gene clusters. The log rank test showed an overall *p* = 0.039. **F** The fraction of tumor-infiltrating immune cells, immune score and stromal score in three gene clusters. The statistical difference of three gene clusters was compared by the Kruskal–Wallis test (^*^p < 0.05, ^**^p < 0.01, ^***^p < 0.001, ^ns^*p* > 0.05)

To prepare for the establishment of ICI scores and visualize the heat map of DEGs between ICI subtypes showing patterns, we used the R package "Limma" to analyze the DEGs among the three ICI subtypes at the genetic level, and a total of 1041 DEGs were obtained (Supplementary Table 6). Specific to the subsequent analysis, TCGA datasets with complete clinical information were primarily analyzed. By unsupervised clustering of these DEGs, the TCGA sample was re-divided into three gene clusters (i.e., A, B and C) (Fig. 2A) (Supplementary Table 7). We define the DEGs that are positively related to gene clusters as gene signature A, and the remaining DEGs as gene signature B (Supplementary Table 8). Moreover, GO analysis was conducted on these two gene signatures, and gene signature A was found to be enriched in the regulation of immune response processes (Fig. 2B), and gene signature B was enriched in cell cycle processes (Fig. 2C) (Supplementary Table 9). The heat map was drawn to visualize the expression of DEGs in different ICI clusters and gene clusters (Fig. 2D).

In addition, we conducted a prognostic analysis on these three gene clusters, and patients in gene cluster C were found to have a better prognosis (*p* = 0.039) (Fig. 2E). After analyzing the ICI in different gene clusters, as indicated from the results, gene cluster C was marked by the high infiltration of naive B cells, Plasma cells, resting memory CD4 + T cells, activated DCs, activated mast cells and immune score. It was also marked by low infiltration of activated memory CD4 + T cells, M0 macrophages, M1 macrophages, M2 macrophages and stromal score (Fig. 2F). The infiltration characteristics of gene cluster C with a better prognosis were almost opposed to the mentioned results of ICI cluster B with a worse prognosis.

### Construction of ICI score

**Fig. 3:**
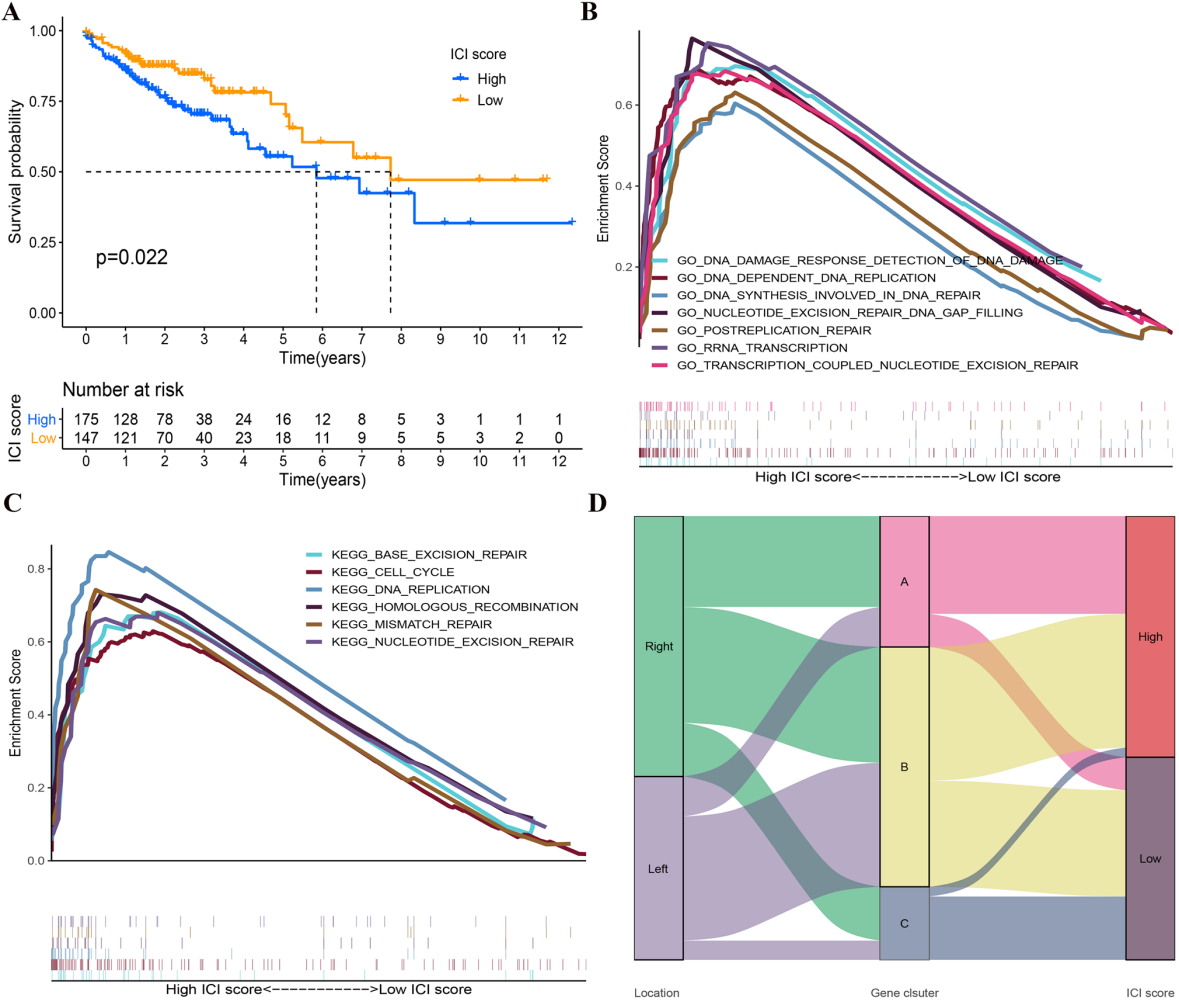
**| A** Kaplan–Meier curves of overall survival in HSG and LSG. The log rank test showed an overall *p* = 0.022. **B** GO-related GSEA showed DNA damage response detection, synthesis involved in DNA repair, postreplication repair, etc. were significantly enriched in the HSG. **C** KEGG-related GSEA showed base excision repair, cell cycle, mismatch repair, etc. were significantly enriched in the HSG. **D** The Sankey diagram showed the distribution of patients with primary tumor sites, gene clusters and ICI scores

To quantify the ICI landscape of patients in LCC and RCC and facilitate the identification of key genes, after the dimensionality reduction in gene signature A and B, PCA was used to compute the aggregate score of feature genes from gene signature A and B, respectively. We obtained the sum of scores and defined them as ICI scores. After the optimal cut-off value was obtained by R package "maxstat," all TCGA patients were stratified into two groups with high or low ICI scores. As indicated from the prognostic analysis, the prognosis of the LSG was better than that of HSG (*p* = 0.022) (Fig. 3A). Then, the univariate and multivariate COX was performed by the combination of ICI score groups with clinical information, including age, gender, tumor node metastasis classification and primary therapeutic modalities. The results indicated that the ICI score group was an independent factor affecting the prognosis (Supplementary Fig. 2A and 2B). To verify the predictive effect of ICI score in Imvigor210 cohort, the ICI score was calculated and the prognostic curve was plotted. The results showed that the ICI score has potential to predict clinical prognosis in the different cohort (*p* = 0.031) (Fig. 5E).

In addition, GO- and KEGG-related GSEA revealed that cell cycle progression, DNA transcription, replication and repair processes were significantly enriched IN THE HSG (Fig. 3B and C).

Subsequently, a Sankey diagram was drawn to show the distribution of patients with tumor sites, gene clusters and ICI scores (Fig. 3D). As indicated by the figure, LCC and RCC were stratified into three gene clusters, and most samples in gene cluster A with worse prognosis belonged to HSG. Moreover, the samples in gene cluster B exhibited an even distribution in HSG and LSG, while the samples in gene cluster C with a better prognosis were mostly distributed in LSG. It was proved that prognostic results obtained by different clustering modes were consistent.

### The role of ICI scores in the prediction of therapeutic benefits

**Fig. 4:**
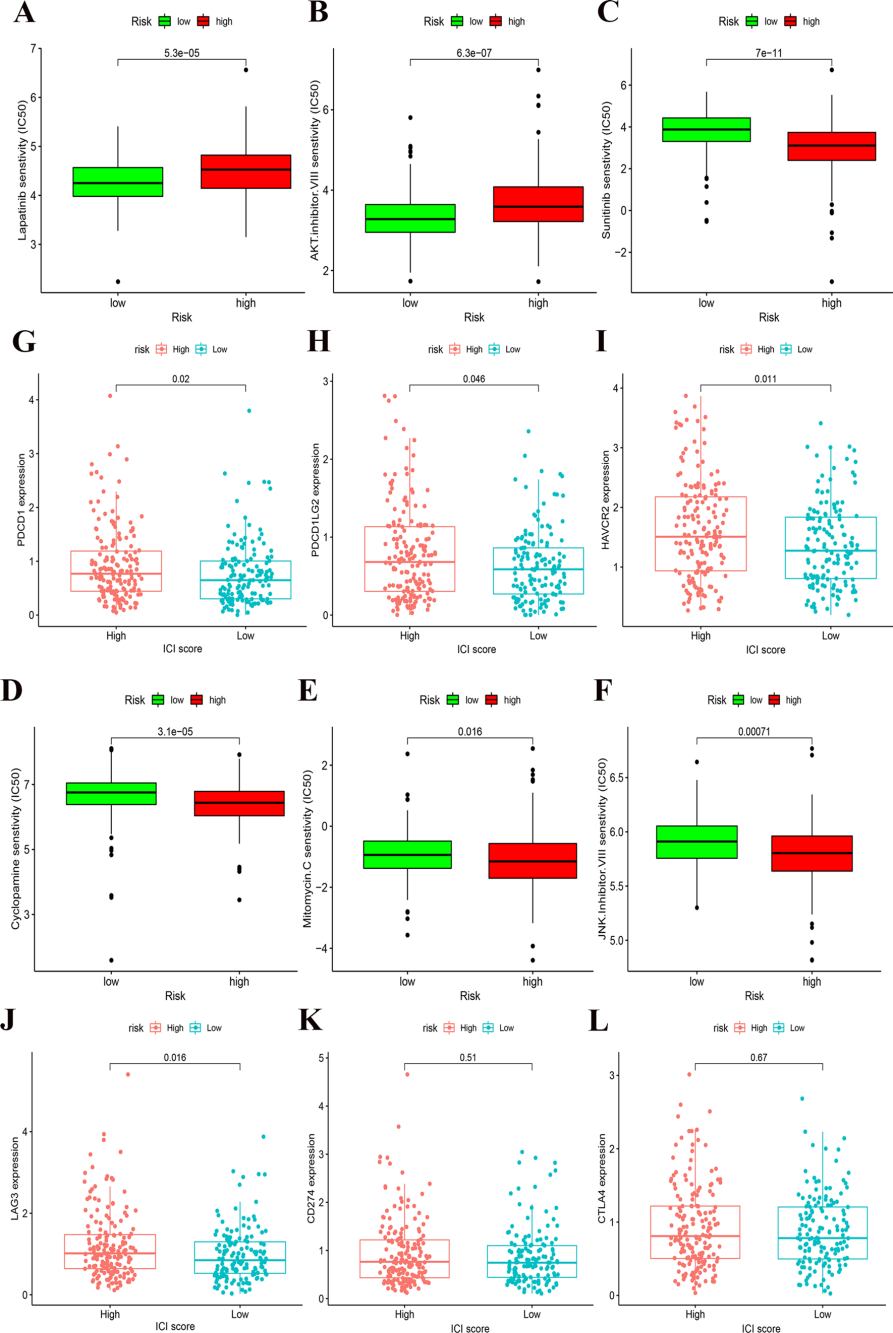
**| A–F** The sensitivity difference of multiple targeted inhibitors in HSG and LSG. In Lapatinib (**A**) and AKT inhibitor VIII (**B**), the median IC50 of LSG was significantly lower than that of HSG (all *p* < 0.05). In Sunitinib (**C**), Cyclopamine (**D**), Mitomycin.C (**A**) and JNK Inhibitor VIII (**F**), HSG had the significantly lower median IC50 than LSG (all *p* < 0.05). (**G**–**I**) The difference of the expression of immunosuppressive checkpoints in HSG and LSG. The expressions of PDCD1 (**G**), PDCD1LG2 (**H**), HAVCR2 (**I**) and LAG3 (**J**) were higher in HSG than those in LSG (all *p* < 0.05). The statistical difference of HSG and LSG was compared by the Wilcoxon test

We analyzed the difference in sensitivity of TIs between the groups. In Lapatinib (epidermal growth factor receptor (EGFR) inhibitor) and AKT inhibitor VIII, the median IC50 of LSG was significantly lower than that of HSG (all *p* < 0.05) (Fig. 4a and b). In Sunitinib (VEGFR2 inhibitor), Cyclopamine (HH signaling inhibitor), Mitomycin.C (DNA synthesis inhibitor) and JNK Inhibitor VIII, HSG had the significantly lower median IC50 than LSG (Fig. 4C–F). For immunosuppressive checkpoints, the expressions of PDCD1, PDCD1LG2, HAVCR2 and LAG3 were higher in HSG than those in LSG (all *p* < 0.05) (Fig. 4G–J), thereby indicating that HSG patients could benefit potentially from immunotherapy.

**Fig. 5:**
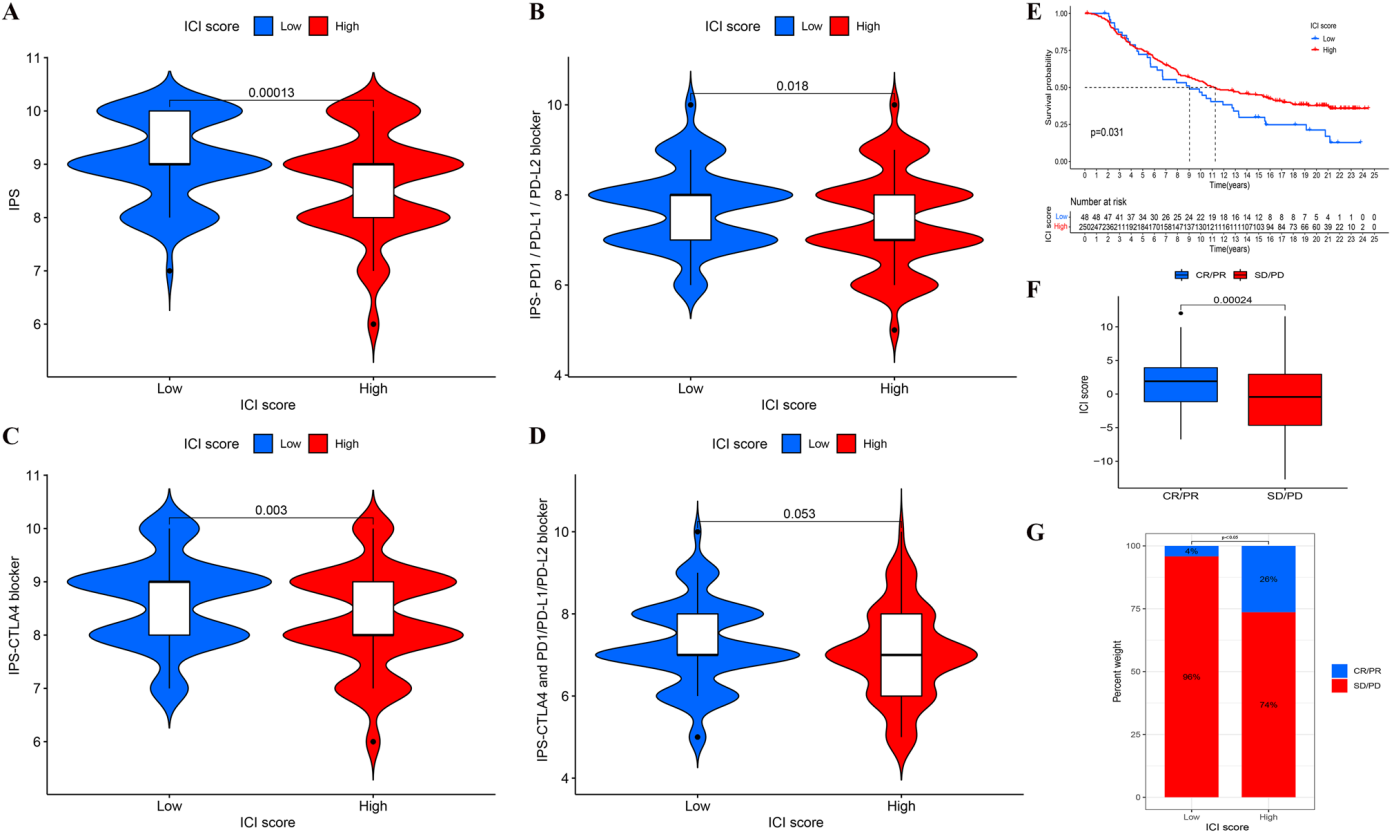
**| **(**A–D**) The relationship between IPS and ICI score groups in LCCs and RCCs patients. The IPS (**A**), IPS-PD1/PD-L1/PD-L2 (**B**) and IPS-CTLA4 (**C**) were significantly different within ICI score groups (all *p* < 0.05). **E** Kaplan–Meier curves of overall survival in the IMvigor210 cohort. The log rank test showed an overall *p* = 0.031. **F** The difference of ICI score between treatment outcome groups (*p* < 0.001). **G** Proportion of patients with different treatment outcomes in HSG and LSG. The proportion of CR/PR patients in HSG was significantly higher than that in LSG (*p* < 0.05). The statistical difference above was compared by the Wilcoxon test

Meanwhile, we used two methods to verify the ability of ICI scores in prediction of immunotherapeutic benefits. Recent studies have reported the role of IPS based on immunogenicity in predicting the immunotherapy response of melanoma patients. We analyzed the relationship of IPS between HSG and LSG. We used IPS, IPS- PD1/PD-L1/PD-L2, IPS-CTLA4 and IPS- PD1/PD-L1/PD-L2 + CTLA4 to evaluate the potential of ICI scores application. The IPS, IPS-PD1/PD-L1/PD-L2 and IPS-CTLA4 were significantly different within ICI score groups (all *p* < 0.05) (Fig. 5A–C). The ICI score in complete response (CR)/partial response (PR) group was significantly higher than that in SD (stable disease)/PD (progressive disease) group (*p* < 0.001) (Fig. 5F), and the proportion of CR/PR patients in HSG was significantly higher than that in LSG (*p* < 0.05) (Fig. 5G). Overall, the ICI score established by us has great potential in predicting prognosis and immunotherapeutic benefits.

### Correlation between ICI score and TMB

As suggested from the enrichment analysis of the gene signatures, they were enriched in the regulation of cell cycle progression. Moreover, GSEA showed that DNA damage repair related pathways were significantly enriched in HSG. According to existing studies, DNA damage and repair abnormalities are directly related to genome instability. Accordingly, we analyzed the correlation between TMB and ICI score in HSG and LSG, respectively.

**Fig. 6:**
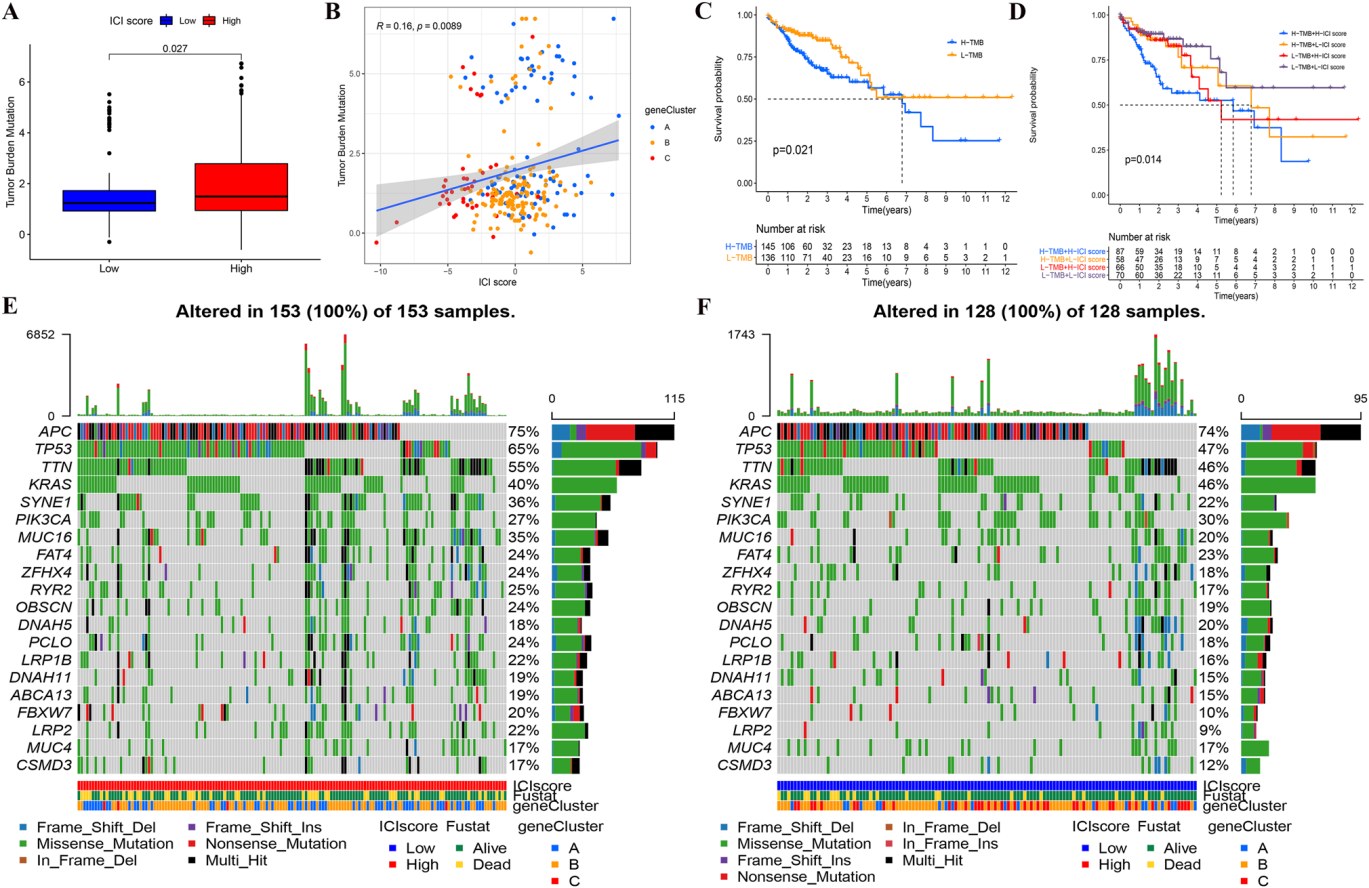
**A** The TMB of HSG was significantly higher than that of LSG. Wilcoxon test, Wilcoxon test, *p* = 0.027. **B** The scatterplots depicted the positive correlation between ICI scores and TMB. The Spearman correlation between ICI scores and TMB was 0.16 (*p* = 0.0089). **C** Kaplan–Meier curves of overall survival in different TMB subgroups. Log rank test, *p* = 0.021. **D** Kaplan–Meier curves of overall survival stratified by both TMB and ICI scores. Log rank test, *p* = 0.014. **E**–**F** The waterfall diagram showed the top 20 driver genes exhibiting the highest mutation frequency in HSG (**E**) and LSG (**F**)

First, the difference of TMB between HSG and LSG was analyzed. As indicated from the results, the TMB of HSG was significantly higher than that of LSG (*p* = 0.027) (Fig. 6a). As suggested from different studies, high TMB led to poor prognosis in considerable cancers [45]. In this study, the results were consistent with them. According to the correlation analysis of ICI score and TMB, we found that there was a significant positive correlation between them (Spearman coefficient: *r* = 0.16, *p* = 0.0089). With the increase in the ICI score, the distribution of gene clusters changed obviously. The samples belonging to gene cluster C with a better prognosis were mainly distributed at the bottom left-hand side (TMB and ICI score were both lower) (Fig. 6b).

Next, we separately analyzed the TMB impact on prognosis. As mentioned previously, patients were stratified into discrete TMB subgroups. As indicated from the results, the prognosis of high-TMB group was worse than that of low-TMB group (*p* = 0.021) (Fig. 6C). Given the synergistic effect of TMB and ICI scores, their effect on prognostic stratification was evaluated. As indicated from the results, TMB status did not interference the predictive ability of ICI score. The survival difference of ICI score subtypes was significant in both high- and low-TMB groups (*p* = 0.014) (Fig. 6D). On the whole, ICI score might act as a prognostic indicator of LCC and RCC that is independent of TMB and can effectively predict TMB and sensitivity of treatment.

Furthermore, we evaluated the differences in driver genes of somatic variation between HSG and LSG. By using waterfall diagram, the top 20 driver genes exhibiting the highest mutation frequency were plotted. It was therefore suggested that the mutation frequency of these genes in HSG was generally higher than that in LSG (Fig. 6E and F). As revealed from the analysis of the mutation annotation files, the mutation frequency of many genes, including TP53 (i.e., tumor suppressor gene) and MSH6 (i.e., mismatch repair-associated genes), was significantly different in HSG and LSG (Supplementary Table 10). The mentioned results might provide novel ideas for investigating targeted therapy and immunotherapy from the aspects of ICI composition and gene mutation.

### Identification of key genes based on ICI score

To screen out key genes from the DEGs, a gene co-expression network was built by using WCGNA to identify important gene modules related to ICI score. By selecting number 4 as the appropriate soft threshold (Supplementary Fig. 3A and 3B), a scale-free co-expression network was built, and 5 modules were obtained (Supplementary Fig. 3C). It was indicated that gray module has the highest correlation with ICI score (Correlation coefficient = − 0.45, *p* < 0.001) (Supplementary Fig. 3D). Thus, we selected the genes in gray module for prognostic analysis. According to the mentioned results, groups with high expressions of CA2, CXCL1, DUOX2, DUOXA2, IER3, PLAC8, TSPAN1 and XDH were related to better prognosis (all p < 0.05) (Supplementary Fig. 4A-4H).

To verify the prognostic capabilities of the mentioned 8 genes identified by ICI score, multivariate COX analysis was conducted, and a prognostic model was built (model 1). In existing studies, CD3 and CD8 acted as indicators to carry out the prognosis of colon cancer [18]. Accordingly, the expressions of CD3D, CD3E, CD3G and CD8 were used to build another prognostic model (model 2). Lastly, the ROC curves were plotted for 3 years (Supplementary Fig. 3E) and 5 years (Supplementary Fig. 3F), respectively, based on the two models. As indicated from the results, model 1 exhibited the better prognostic capability than model 2.

Moreover, we searched for the first neighbor of these genes in the DisNor database. The database consisted of two genes (i.e., CXCL1 and IER3), as well as their direct targets (Supplementary Fig. 5A). The upstream regulators of CXCL1 included SMO, CEBPD, MTA1 and FZD3, and the downstream regulators involved PLCE1, PRKACA, GLI1, GLI2 and GLI3. HNRNPU and MAPK1 were the upstream regulators of IER3, and PPP2R5C was the downstream regulator. The PPI analysis revealed that the complex interaction between genes above and the other 6 genes (Supplementary Fig. 5B).

**Fig. 7:**
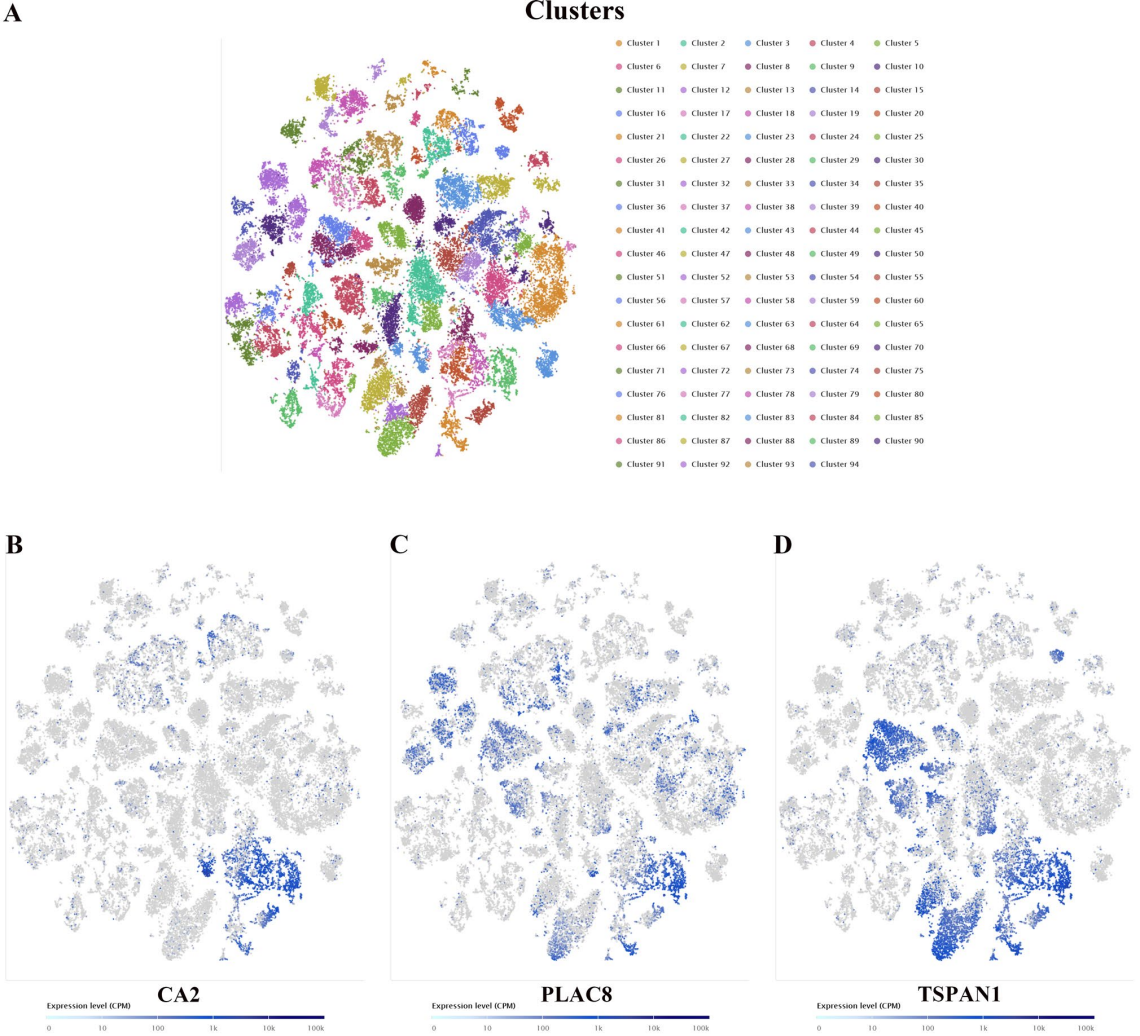
**A** In SCEA database, after setting the suitable parameters (t-SNE perplexity score = 25, *k* value = 94), all colon cancer single cells were clustered into 94 subpopulations according to their expression patterns. **B**–**D** The key genes with specific expression patterns in single-cell level, including CA2 (**B**), PLAC8 (**c**) and TSPAN1 (**D**). They could act as the marker genes in cluster 9

We explored the expression pattern of these genes in single-cell level based on the single-cell sequencing dataset (E-MTAB-8410) in SCEA database [41] (Fig. 7A) (Supplementary Fig. 6A-6E). The detailed characteristics of 9 samples in the dataset were provided in supplementary table 11. By setting the suitable parameters (t-SNE perplexity score = 25, *k* value = 94), we found that the three genes had specific expression characteristics in single-cell level, including CA2, PLAC8 and TSPAN1 (Fig. 7B–D). After the cells were isolated into 94 subpopulations, they could act as the marker genes in cluster 9 based on the analysis results (Supplementary Table 12) (Supplementary Fig. 7) [46]. According to the results provided in the SCEA database, 1153 cells of the total 60,383 cells belong to cluster 9. Cells were colored according to clusters, marker genes, inferred cell types, individual and sampling sites (Supplementary Fig. 8A-C). For the novel cell subpopulation (cluster 9) we discovered, we searched for the cell markers in CellMarker database (http://biocc.hrbmu.edu.cn/CellMarker/) [47] and inferred that they belonged to a subpopulation of intestine epithelial cells. Among the top 20 ranked marker genes in cluster 9, cell markers belonging to epithelial cells included GUCA2A, GUCA2B, CEACAM7, CDHR5 and AQP8 [48]. The GO and KEGG analysis revealed that maker genes in cluster 9 were mainly enriched in cell adhesion, transport of substance related processes, leucocyte transendothelial migration (Supplementary Fig. 9A and 9B). Intestinal epithelial cells can secrete various cytokines and chemokines to modulate host immune responses [49]. To date, a number of studies have found that the immune response at the intestinal epithelium was involved in the origin and development of CC. Cytokines released by epithelial and immune cells play a role in the pathogenesis of colitis-associated cancer [50]. These findings suggest that novel subpopulation of intestine epithelial cells may have a special effect on tumor development and prognosis.

**Fig. 8:**
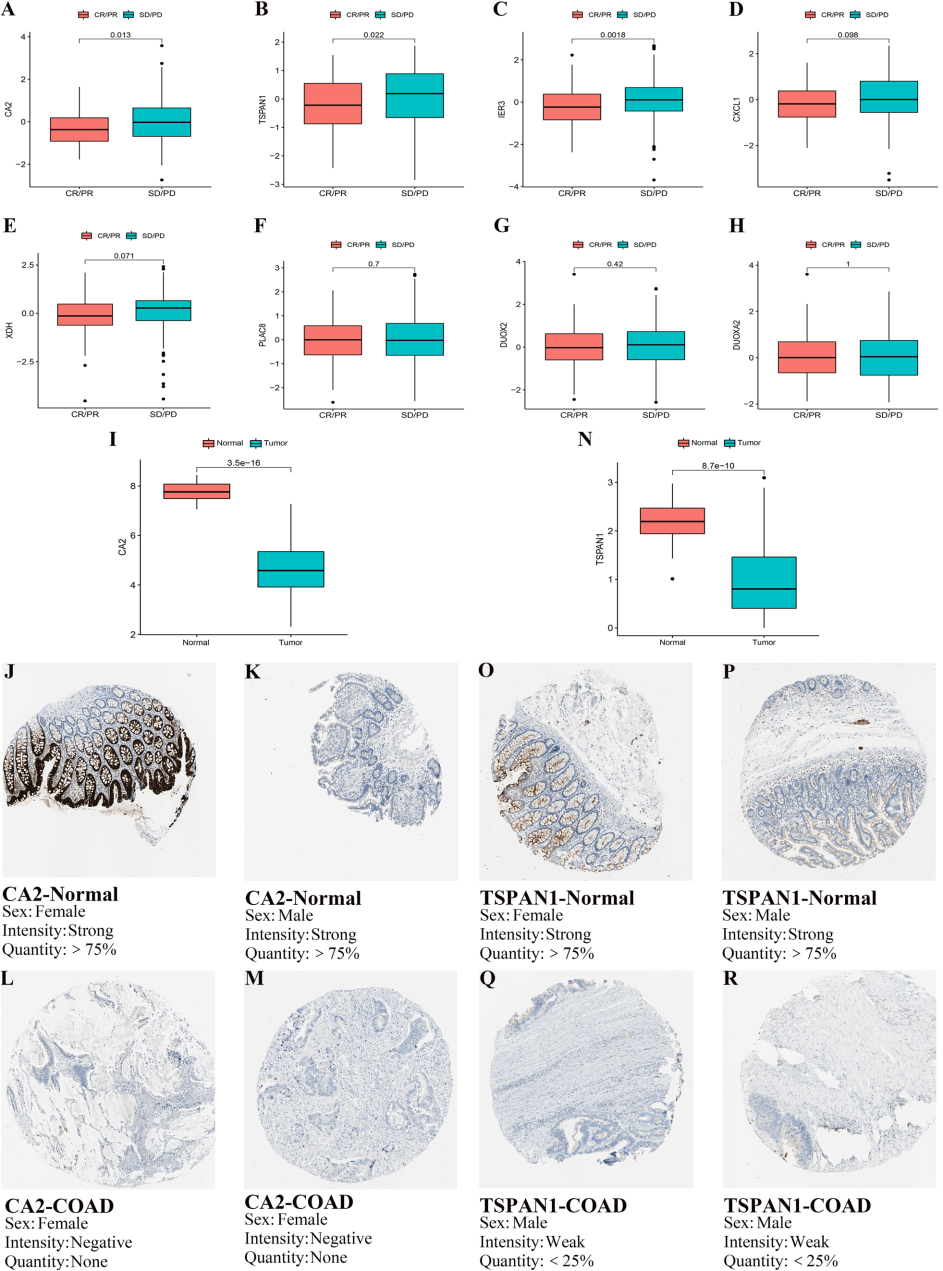
(**A**–**H**) The expression differences of the 8 preliminary screened genes in different treatment outcome groups. The expressions of CA2 (**A**), TSPAN1 (**B**) and IER3 (**C**) were significantly different in treatment outcome groups (all *p* < 0.05). (**i** and **N**) The expression of CA2 (**I**) and TSPAN1 (**N**) were significantly different between cancer tissues and normal tissues at protein level (all *p* < 0.05). The statistical difference above was compared by the Wilcoxon test. (**J**–**M**) Immunohistochemical staining for the key genes CA2 in normal tissues (**j** and **K**) and COAD cancer tissues (**L** and **M**). **O**–**R** Immunohistochemical staining for the key genes TSPAN1 in normal tissues (**O** and **P**) and COAD cancer tissues (**Q** and **R**). (Image credit: Human Protein Atlas, images available from v20.1.proteinatlas.org.)

Lastly, we analyzed the expression of mentioned 8 genes in the immunotherapy cohort. The results showed that the expressions of CA2, IER3 and TSPAN1 were significantly different in treatment outcome groups (all *p* < 0.05) (Fig. 8A–C). Meanwhile, we found that the expression of CA2 (Fig. 8I–M) and TSPAN1 (Fig. 8N–R) was significantly different between cancer tissues and normal tissues at protein level (all *p* < 0.05). After a series of screening processes, CA2 and TSPAN1 could be used as potential markers to predict the sensitivity of immunotherapy.

## Discussion

The inhibitory TAIM, characterized by specific immune cell infiltration, has been increasingly proven to be the culprit of immunotherapy resistance and tumor progression [51]. In this study, ICI subtypes and gene subtypes related to prognosis were identified in LCC and RCC. After the intersection of immune cells was taken, this study reported that the ICI features with a better prognosis were marked by high plasma cells, DCs and MCs, low activated memory CD4 + T cells, M0 macrophages, M1 macrophages, as well as M2 macrophages. Specific to colorectal cancer, Maartje indicated that tumor-infiltrating B cells and plasma cells were significantly correlated with a better prognosis [52]. In a multiple cancer analysis, in the presence of both B cells and plasma cells, the prognostic effect of T cells tended to increase [53]. DCs considered to be professional antigen presenting cells are capable of performing powerful immune responses against tumor cells [54]. In addition, DC vaccines combined with other cancer therapies can contribute to efficient cancer therapeutics [55]. MCs, a type of innate immune cells, exert a pro-tumorigenic or anti-tumorigenic effect, which is determined by the type of cancer [56]. In CC, MCs were reported to be able to be recruited by IL-17 produced by regulatory T cells (Tregs), which further facilitates the infiltration of immune cells [57]. However, its impact on the development and prognosis of CC requires in-depth studies. Salman found that CD4 + T cells were highly expressed in CC tissues. The mentioned CD4 + T cells highly expressed the markers of activated Tregs (i.e., Helios and Foxp3). Moreover, the intratumoral CD4 + T cells can up-regulate various inhibitory immune checkpoints (e.g., PD1, CTLA-4, TIM-3 and LAG-3) [58]. Accordingly, activated CD4 + T cells may promote inhibitory TAIM in CC. M2 macrophages suppress the anti-tumor immune response in various manners [59], and M0 macrophages may be related to the distant metastasis and poor prognosis of COAD [60]. In this study, low macrophages infiltrated subtypes were related to a better prognosis. The mentioned ICI features are unique in LCC and RCC and could be applied for predicting prognosis. These immune cells may disturb the balance between immune tolerance and activity via intercellular communication.

By complying with the individual differences in TAIM, the ICI score was set to quantify the ICI landscape. The DEGs related to ICI subtypes were found to be significantly enriched in immune regulation, cell cycle, as well as DNA damage repair pathways. Besides, the identical result was identified in the GSEA of HSG and LSG. As reported by recent studies, the genomic instability could produce immune-responsive phenotypes that impact the immune response and immunotherapy [61]. In LCC and RCC, however, the relationship between them has been rarely clarified. Thus, the correlation between TMB and ICI scores was analyzed in depth, and significant differences were identified in the mutation frequency of multiple genes between HSG and LSG. In addition, a significant positive correlation was found between the ICI score and TMB. For this reason, the genomic instability could induce the difference of ICI. A wide range of Tis were reported to show significant differences in sensitivity between HSG and LSG (e.g., VEGFR2 and HH inhibitors). HH have been confirmed as the stemness-related signals of cancer stem cells (CSCs) [62, 63]. CSCs could induce tumor recurrence and trigger low responses to immunotherapy and drug resistance [64]. Accordingly, it has great potential to develop TIs, and the ICI scores here can be referenced for their applications in colon cancer. Moreover, the gene expression of immunosuppressive checkpoints was found to be significantly different in two groups. In cancers with overexpression of immunosuppressive checkpoints, immune checkpoint inhibitors were suggested to be effective to enhance anti-tumor effect and clinical impact of T cell [65]. Thus, this study speculated that patients in the HSG can benefit from immunotherapy. However, the effect of immunotherapy in patients with colon cancer still needs further study. Furthermore, stratified prognostic analysis revealed that the value of ICI scores on prognosis was independent of TMB in LCC and RCC, indicating that ICI scores and TMB influence the prognosis from distinct aspects of immunobiology.

Limitations are as follows: we require additional experiments to investigate the specific function of these prognostic key genes. And more independent immunotherapy cohorts are required for validation to ensure the accuracy and robustness of the ICI scores.

In summary, the ICI landscape of LCC and RCC was comprehensively stratified and quantified, and a novel and effective method was developed to evaluate the prognosis and assess treatment sensitivity. The identification of ICI subtypes will help gain insights into the heterogeneity in LCC and RCC. The ICI scores and the key genes CA2 and TSPAN1 could serve as an effective biomarker to predict prognosis and immunotherapy response. Moreover, CA2 and TSPAN1 were found to act as marker genes in a novel subpopulation. The findings of the present study should be validated based on clinical trials in a larger cohort.

## Supplementary Information

Below is the link to the electronic supplementary material.

Supplementary Figure 1 | Overall flowchart of this study (TIF 422 kb)

Supplementary Figure 2 | (A-B) The ICI-score groups was an independent factor affecting the prognosis analyzed by the univariate- (A) and multivariate- COX (B) (all p<0.05). (TIF 397 kb)

Supplementary Figure 3 | (A-B) To achieve a scale-free co-expression network, the power index=4 was chosen as the appropriate soft threshold. (C) The branches of the dendrogram correspond to 5 different gene modules. (D) The correlation between the gene modules and ICI scores. Each cell contains corresponding correlation coefficient and p-value. (E-F) The ROC curves plotted for 3 years (E) and 5 years (F), respectively, based on the two models. Model 1 based on genes identified by ICI scores exhibited the better capability of prognostic prediction than model 2. (TIF 10237 kb)

supplementary figure 4 | (A-H) Kaplan-Meier curves of overall survival in 8 key genes preliminary identified by ICI scores, including CA2 (A), CXCL1 (B), DUOX2 (C), DUOXA2 (D), IER3 (E), PLAC8 (F), TSPAN1 (G) and XDH (H). Log rank test, all p<0.05. (TIF 1085 kb)

Supplementary Figure 5 | (A) The causal interaction of key gene analysis in DisNor. The database consisted of two genes identified by ICI scores (i.e., CXCL1 and IER3), as well as their direct targets. (B) The PPI analyses between key genes and directly interacted genes identified by DisNor. The thickness of the solid line represents the strength of the relationship. (TIF 712 kb)

Supplementary Figure 6 | (A-E) The expression patterns of preliminary screened genes in single-cell level, including CXCL1 (A), DUOX2 (B), DUOXA2 (C), IER3 (D) and XDH (E). The expression patterns of these genes were not specific enough to represent a cell population in single-cell level. (TIF 6977 kb)

Supplementary Figure 7 | The heatmap of marker genes in 94 clusters. We displayed the top 15 ranked marker genes in each cluster. The color of each square indicates the average gene expression (white to blue). (TIF 4779 kb)

Supplementary Figure 8 | (A-C) Cells were colored according to clusters, marker genes, inferred cell types (A), individual (B) and sampling sites (C). For the novel cell subpopulation (pink cells marked by red circle), we inferred that they belonged to a subpopulation of intestine epithelial cells by CellMarker database. (TIF 9783 kb)

Supplementary Figure 9 | (A) GO analysis on marker genes in cluster 9. (B) KEGG analysis on marker genes in cluster 9. (TIF 1158 kb)

Supplementary file10 (XLS 1164 kb)

## Abbreviations

AM: Adjacency matrix
CC: Colon carcinoma
COAD: Colon adenocarcinoma
COX: Cox proportional hazard regression analysis
CSCs: Cancer stem cells
DCs: Dendritic cells
DEGs: Differentially expressed genes
GEO: Gene expression omnibus
GO: Gene ontology
GSEA: Gene set enrichment analysis
HH: Hedgehog
HSG: High-ICI score groups
ICI: Immune cell infiltration
IC50: Concentration causing 50% reduction growth
LCCs: Left-sided colon carcinomas
LSG: Low-ICI score groups
MCs: Mast cells
PCA: Principal component analysis
PPI: Protein–protein interaction
RCCs: Right-sided colon carcinomas
SCEA: The single cell expression atlas
T-SNE: T-distributed stochastic neighbor embedding
TAIM: Tumor-associated immune microenvironment
TCGA: The cancer genome atlas
TIs: Targeted inhibitors
TMB: Tumor mutation burden
Tregs: Regulatory T cells
WGCNA: Weighted gene co-expression network analysis
