# Supplementary figures and images for: Identification and quantification of immune infiltration landscape on therapy and prognosis in left- and right-sided colon cancer
Source: Cancer Immunol Immunother. 2021 Oct 16;71(6):1313–30. doi: 10.1007/s00262-021-03076-2 (PMC9122887; doi:10.1007/s00262-021-03076-2)

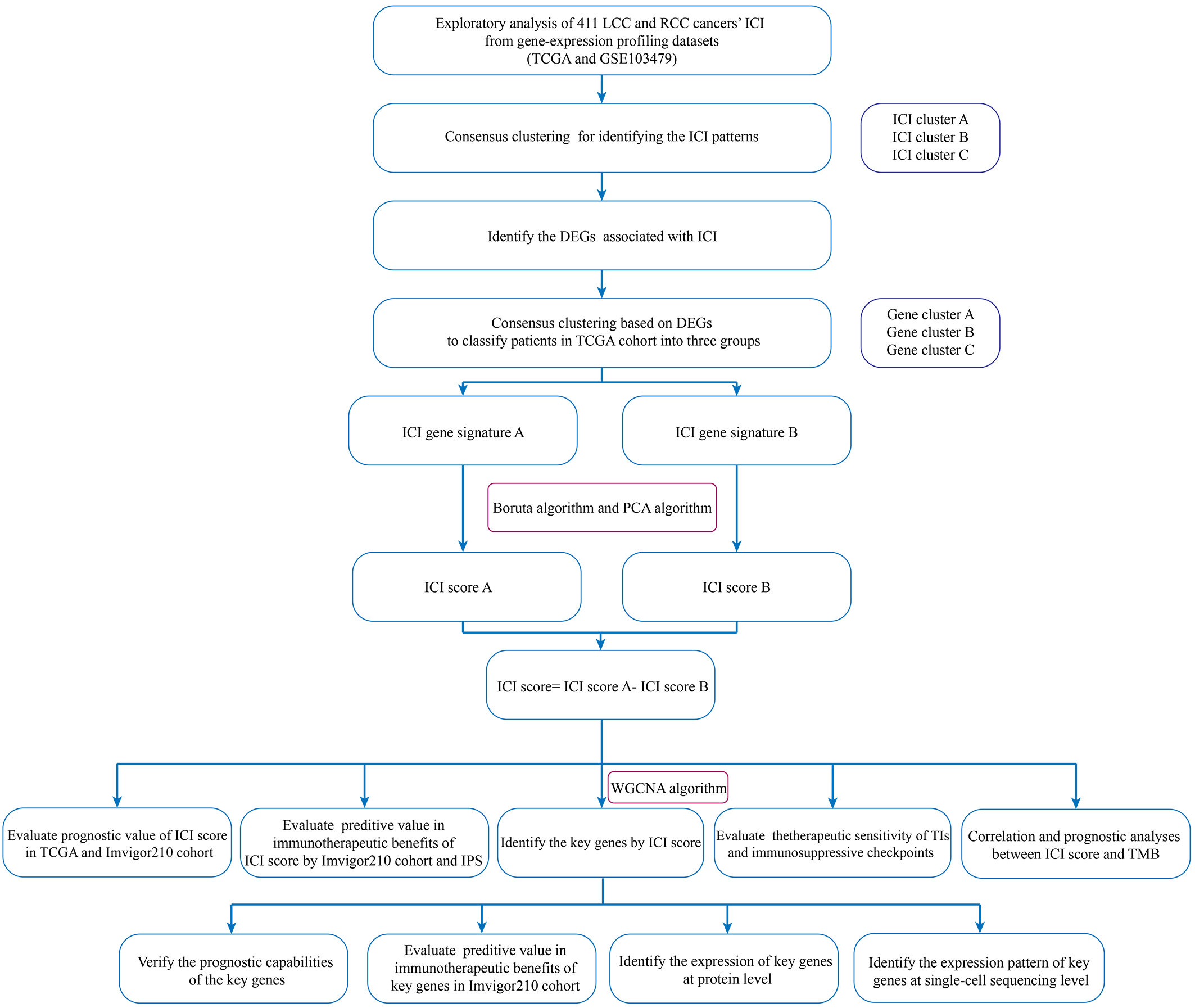

Supplement: Supplementary file 1 — Supplementary Figure 1 | Overall flowchart of this study (TIF 422 kb) [file 262_2021_3076_MOESM1_ESM.tif]

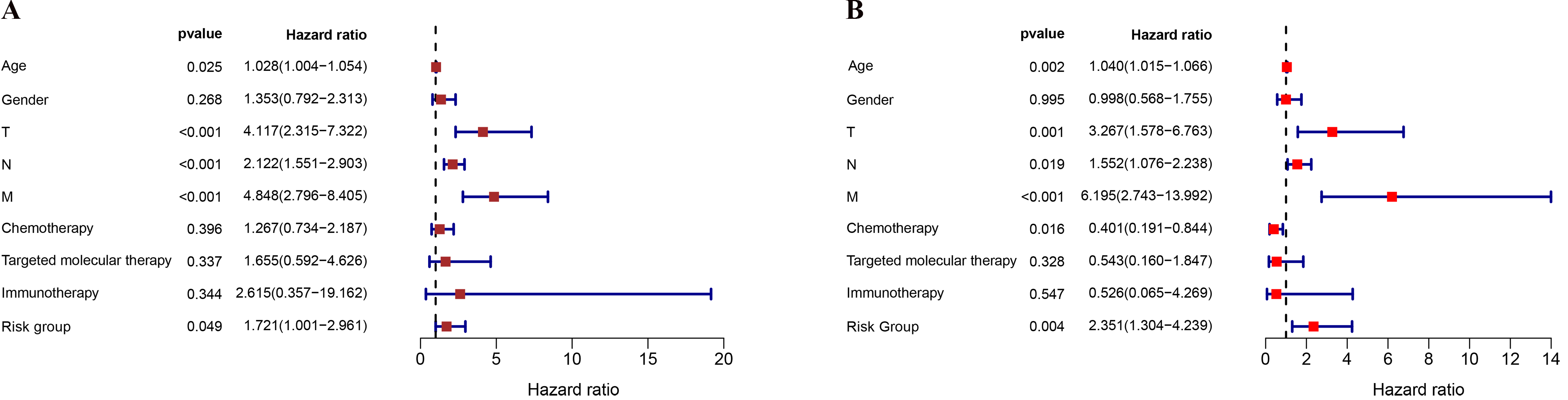

Supplement: Supplementary file 2 — Supplementary Figure 2 | (A-B) The ICI-score groups was an independent factor affecting the prognosis analyzed by the univariate- (A) and multivariate- COX (B) (all p<0.05). (TIF 397 kb) [file 262_2021_3076_MOESM2_ESM.tif]

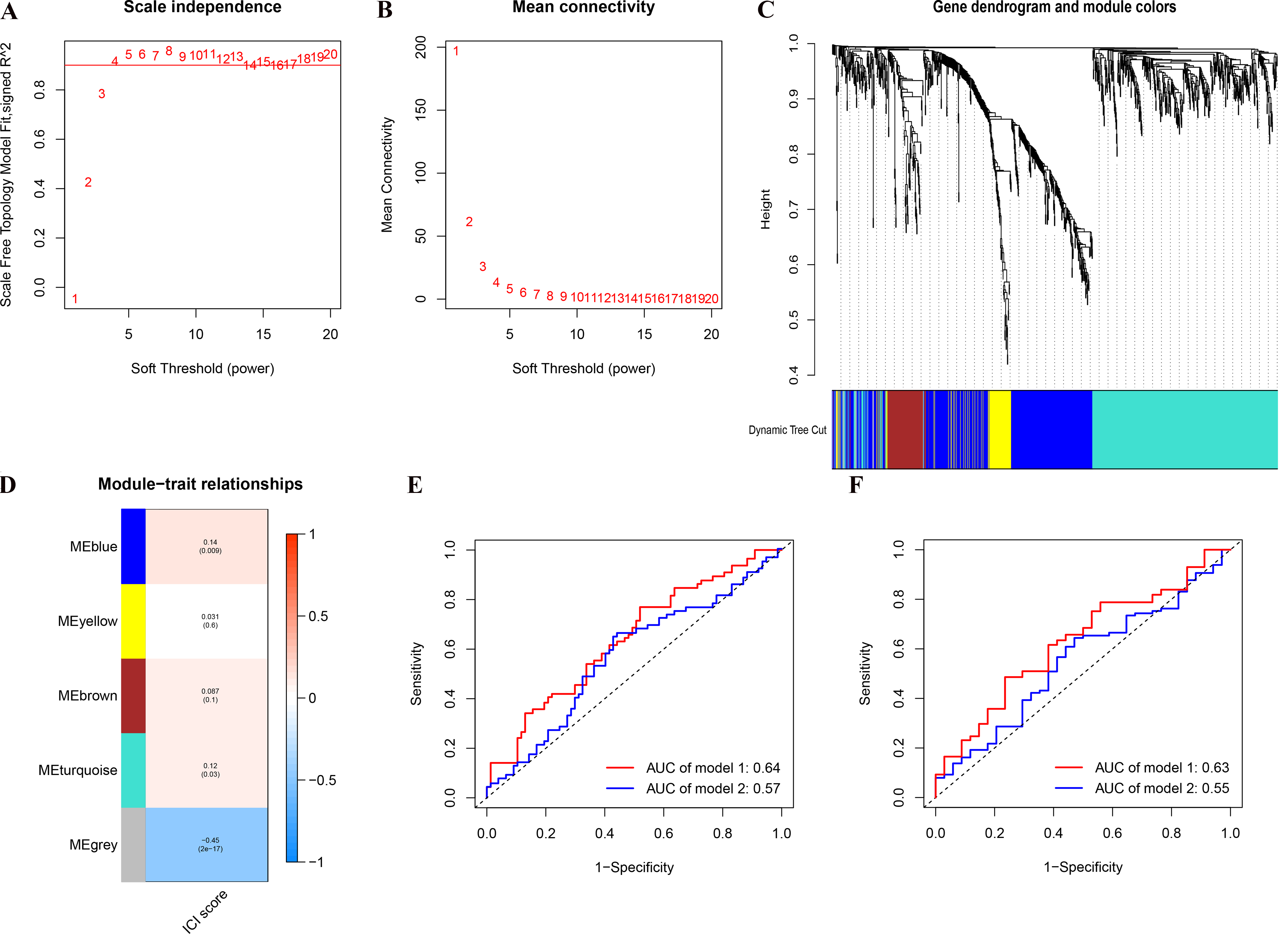

Supplement: Supplementary file 3 — Supplementary Figure 3 | (A-B) To achieve a scale-free co-expression network, the power index=4 was chosen as the appropriate soft threshold. (C) The branches of the dendrogram correspond to 5 different gene modules. (D) The correlation between the gene modules and ICI scores. Each cell contains corresponding correlation coefficient and p-value. (E-F) The ROC curves plotted for 3 years (E) and 5 years (F), respectively, based on the two models. Model 1 based on genes identified by ICI scores exhibited the better capability of prognostic prediction than model 2. (TIF 10237 kb) [file 262_2021_3076_MOESM3_ESM.tif]

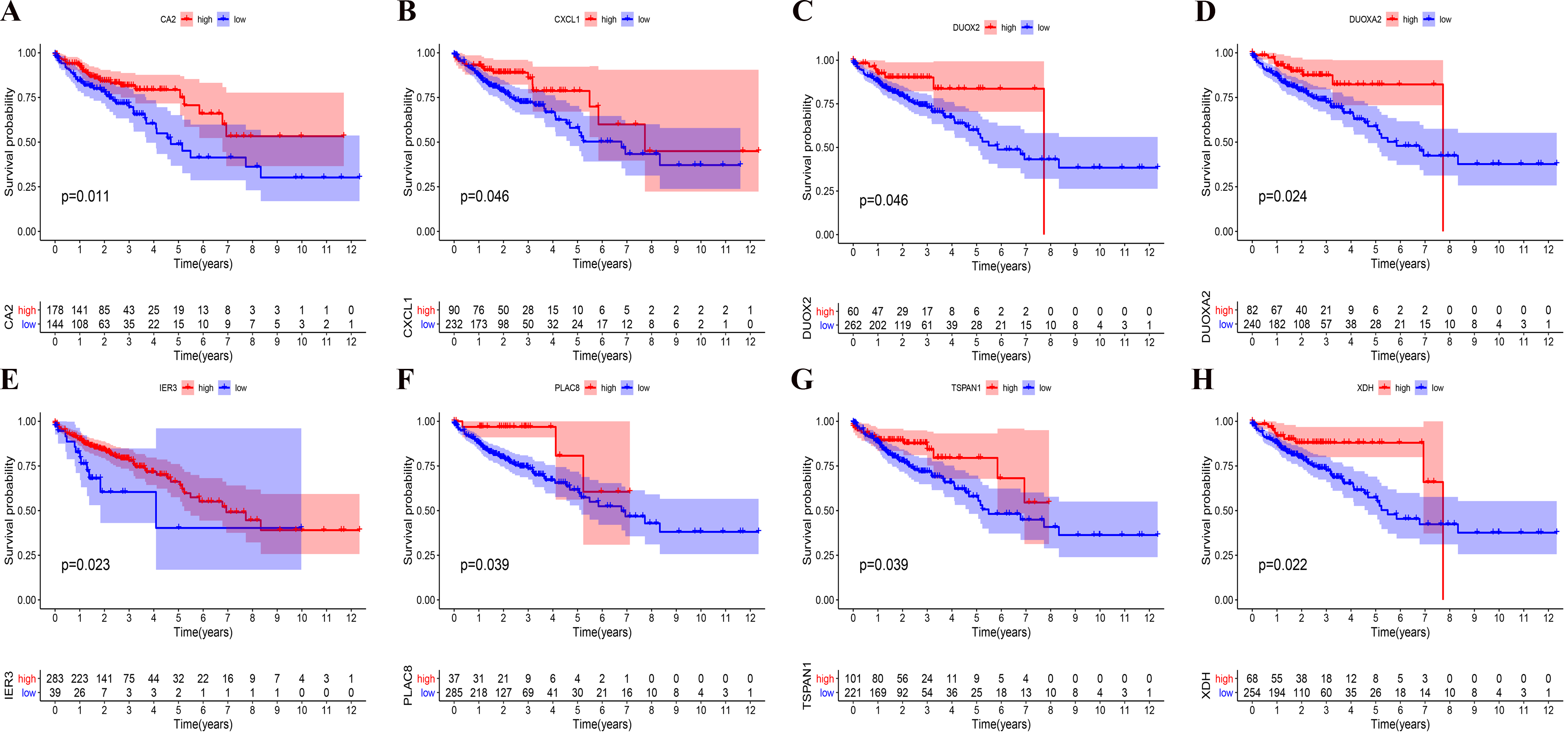

Supplement: Supplementary file 4 — supplementary figure 4 | (A-H) Kaplan-Meier curves of overall survival in 8 key genes preliminary identified by ICI scores, including CA2 (A), CXCL1 (B), DUOX2 (C), DUOXA2 (D), IER3 (E), PLAC8 (F), TSPAN1 (G) and XDH (H). Log rank test, all p<0.05. (TIF 1085 kb) [file 262_2021_3076_MOESM4_ESM.tif]

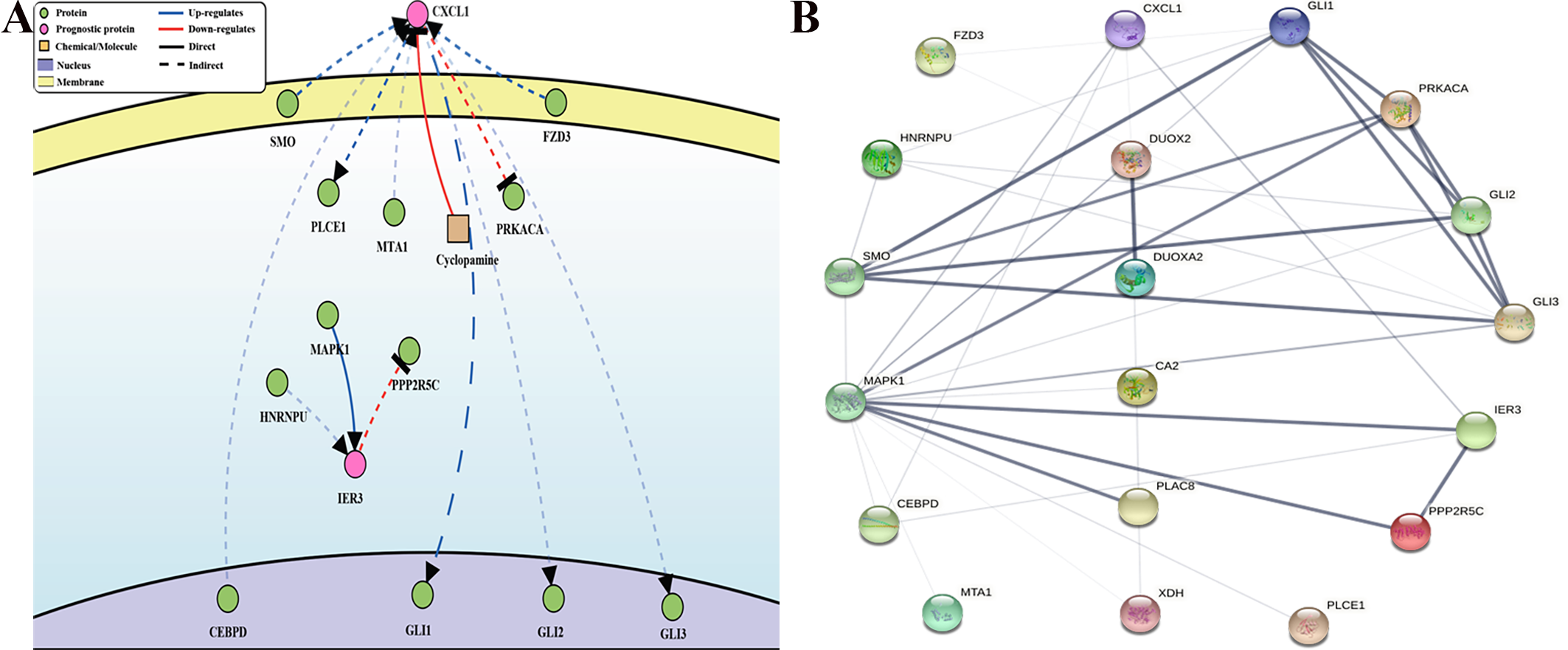

Supplement: Supplementary file 5 — Supplementary Figure 5 | (A) The causal interaction of key gene analysis in DisNor. The database consisted of two genes identified by ICI scores (i.e., CXCL1 and IER3), as well as their direct targets. (B) The PPI analyses between key genes and directly interacted genes identified by DisNor. The thickness of the solid line represents the strength of the relationship. (TIF 712 kb) [file 262_2021_3076_MOESM5_ESM.tif]

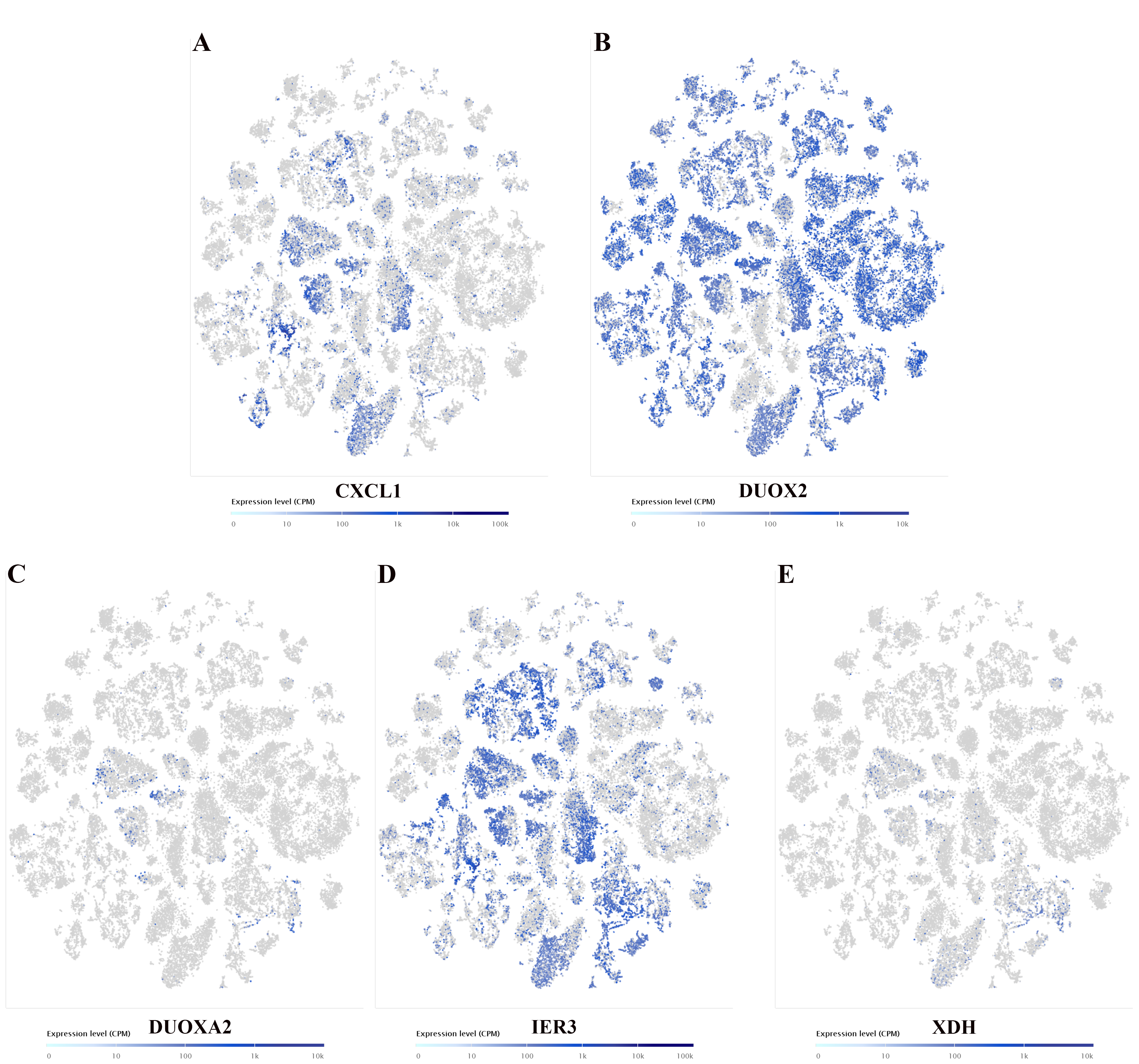

Supplement: Supplementary file 6 — Supplementary Figure 6 | (A-E) The expression patterns of preliminary screened genes in single-cell level, including CXCL1 (A), DUOX2 (B), DUOXA2 (C), IER3 (D) and XDH (E). The expression patterns of these genes were not specific enough to represent a cell population in single-cell level. (TIF 6977 kb) [file 262_2021_3076_MOESM6_ESM.tif]

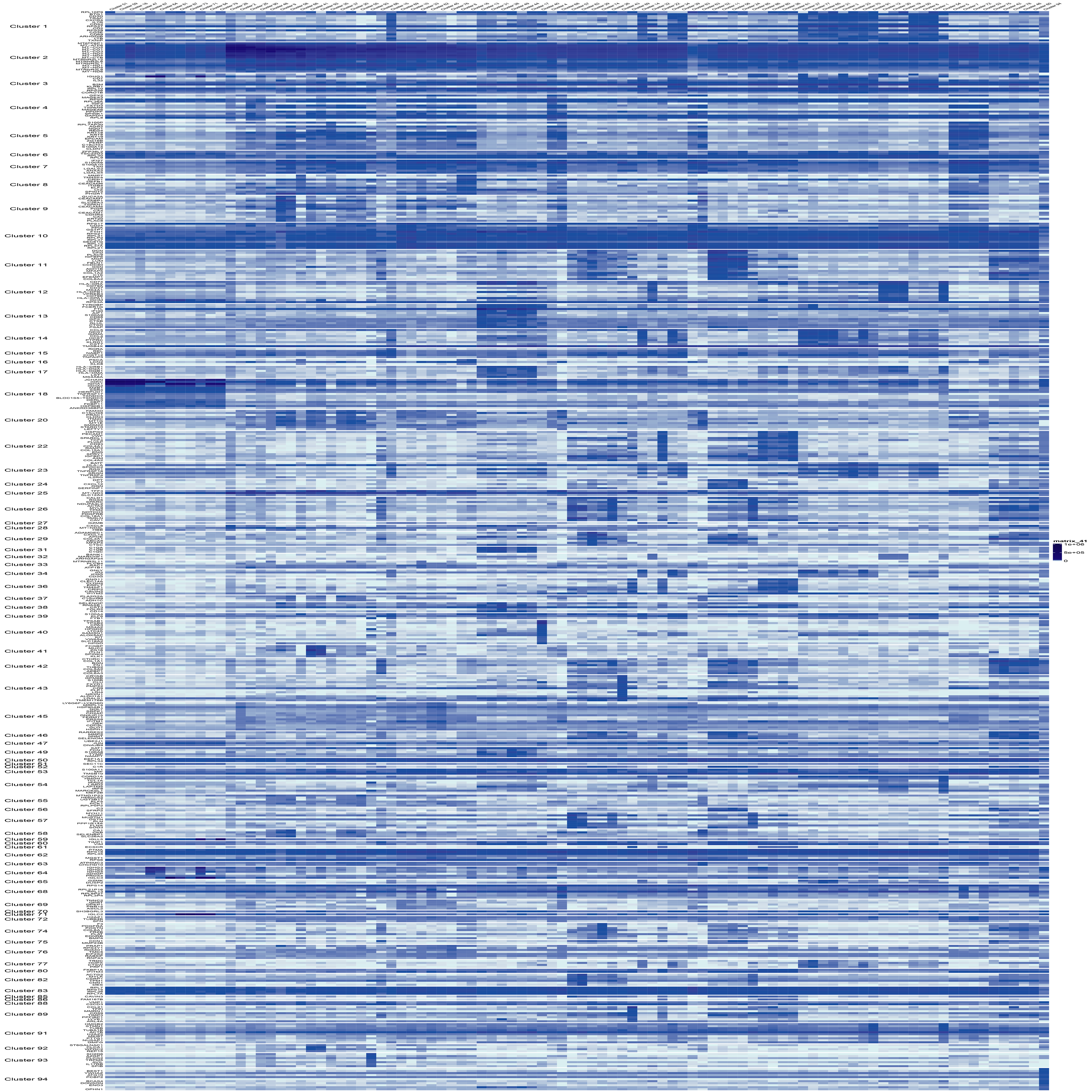

Supplement: Supplementary file 7 — Supplementary Figure 7 | The heatmap of marker genes in 94 clusters. We displayed the top 15 ranked marker genes in each cluster. The color of each square indicates the average gene expression (white to blue). (TIF 4779 kb) [file 262_2021_3076_MOESM7_ESM.tif]

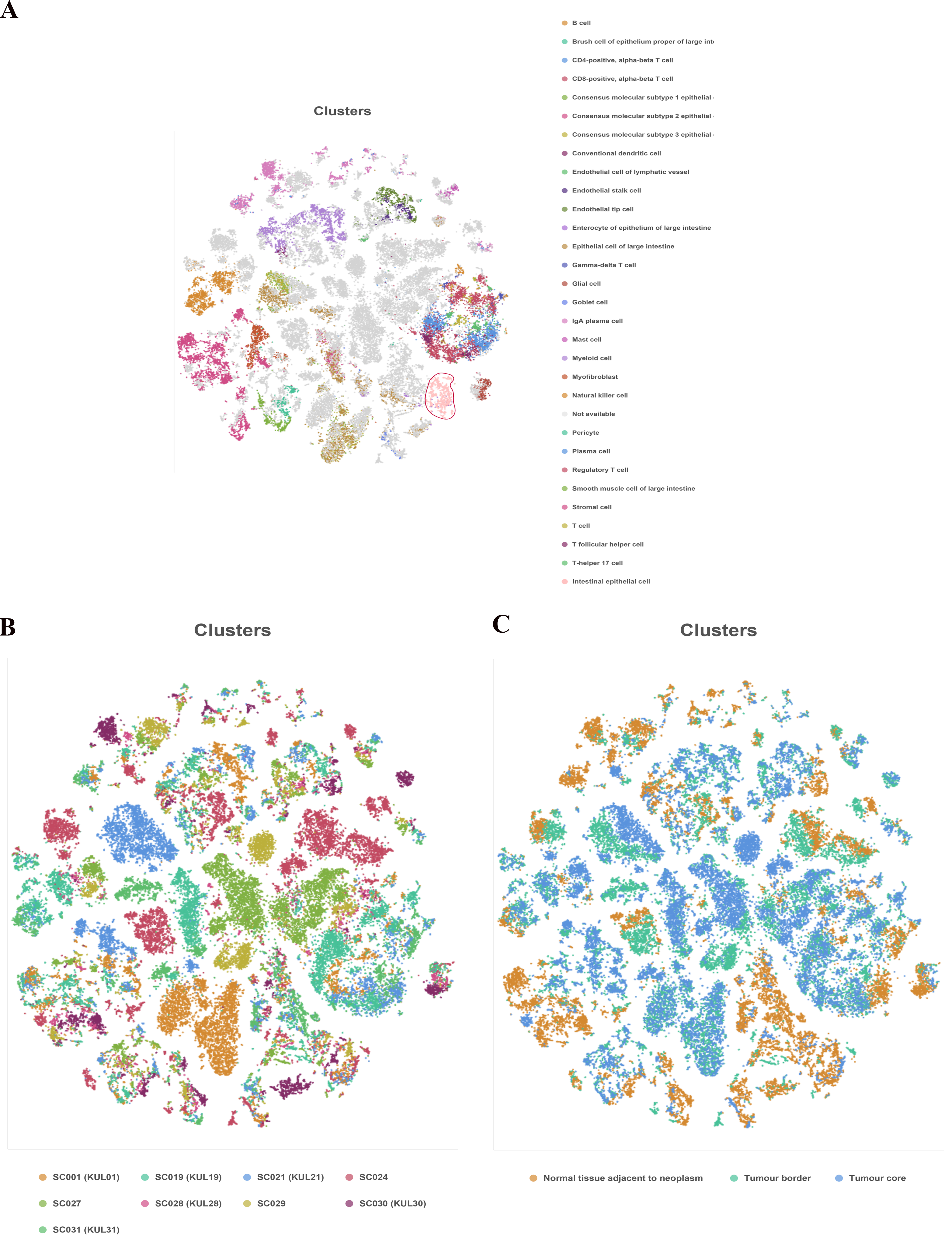

Supplement: Supplementary file 8 — Supplementary Figure 8 | (A-C) Cells were colored according to clusters, marker genes, inferred cell types (A), individual (B) and sampling sites (C). For the novel cell subpopulation (pink cells marked by red circle), we inferred that they belonged to a subpopulation of intestine epithelial cells by CellMarker database. (TIF 9783 kb) [file 262_2021_3076_MOESM8_ESM.tif]

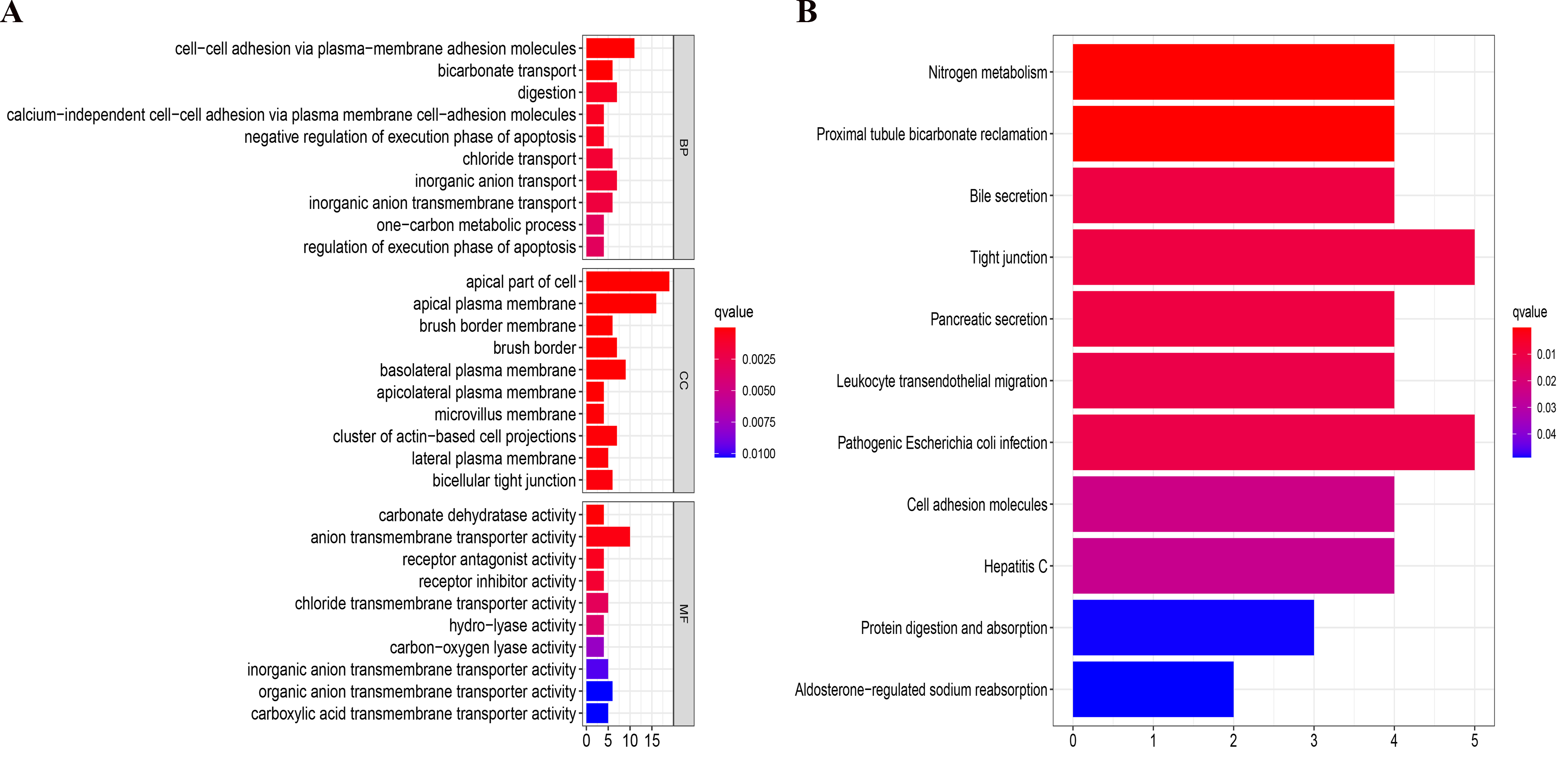

Supplement: Supplementary file 9 — Supplementary Figure 9 | (A) GO analysis on marker genes in cluster 9. (B) KEGG analysis on marker genes in cluster 9. (TIF 1158 kb) [file 262_2021_3076_MOESM9_ESM.tif]
